# Quantifications of CD4+ T-Lymphocytes levels in adult sickle cell patients: Examining immunological vulnerability and Vaso-Occlusive crises in a low-resource setting, northwestern Nigeria

**DOI:** 10.1093/oxfimm/iqaf013

**Published:** 2026-01-22

**Authors:** Abubakar Babangida Usman, Abubakar Hassan Gulma, Kabir Magaji Hamid, Adamu Muhammad Ibrahim, Shuaibu Saidu Musa, Jomar L Aban, Jerico Bautista Ogaya, Kristine Joy Gacutno, Angelica Joyce Gacutno-Evardone, Carina Joane V Barroso, Pearl Irish V De Paz, Mohamed Mustaf Ahmed, Sani Muhammed, Don Eliseo Lucero-Prisno

**Affiliations:** Department of Immunology, School of Medical Laboratory Sciences, Usmanu Danfodiyo University, Sokoto, 840001, Nigeria; Department of Immunology, School of Medical Laboratory Sciences, Usmanu Danfodiyo University, Sokoto, 840001, Nigeria; Department of Immunology, School of Medical Laboratory Sciences, Usmanu Danfodiyo University, Sokoto, 840001, Nigeria; Department of Immunology, School of Medical Laboratory Sciences, Usmanu Danfodiyo University, Sokoto, 840001, Nigeria; School of Global Health, Faculty of Medicine, Chulalongkorn University, Bangkok, 10330, Thailand; Department of Nursing Science, Ahmadu Bello University, Zaria, Kaduna, 210001, Nigeria; College of Education, Don Mariano Marcos Memorial State University, Bacnotan, La Union, 2515, Philippines; Department of Medical Technology, Institute of Health Sciences and Nursing, Far Eastern University, Manila, 1215, Philippines; Center for University Research, University of Makati, Makati City, Makati, 1215, Philippines; Eastern Visayas Medical Center, Leyte, Tacloban City, Leyte, 6500, Philippines; Eastern Visayas Medical Center, Leyte, Tacloban City, Leyte, 6500, Philippines; Remedios Trinidad Romualdez Medical Foundation, Tacloban City, Tacloban City, Leyte, 6500, Philippines; College of Nursing, Bukidnon State University, Malaybalay, Bukidnon, 8700, Philippines; Biliran Province State University, Naval, Biliran, 6543, Philippines; School of Global Health, Faculty of Medicine, Chulalongkorn University, Bangkok, 10330, Thailand; SIMAD University, Mogadishu, Somalia; Department of Health Sciences, School of Medicine, University of Piemonte Orientale, Novara, 15121, Italy; Department of Global Health and Development, London School of Hygiene and Tropical Medicine, WC1E 7HT, United Kingdom; Research and Innovation Office, Biliran Province State University, Biliran Province, Naval, 6560, Philippines; Center for Research and Development, Cebu Normal University, Cebu City, 6000, Philippines

**Keywords:** CD4+, T-lympocyte, Sickle Cell Disease, Sokoto, Nigeria, levels, Northwestern

## Abstract

Introduction: Sickle Cell Disease (SCD) is a chronic genetic disorder that impairs red blood cell function and contributes to recurrent complications, particularly vaso-occlusive crises. Aim: This study evaluates the level of CD4 + T-lymphocyte counts in adult SCD patients in Northwestern Nigeria, assessing their association with clinical states, and epidemiological factors, to better understand immunological vulnerability. Methods: A descriptive cross-sectional study was conducted at Specialist Hospital Sokoto, 45 adult SCD patients were recruited and stratified by clinical status (steady state versus crisis). CD4 + counts were measured via flow cytometry, and data were analyzed for relationships with haemoglobin levels, gender, and age. Results: Patients in vaso-occlusive crisis observed reduction in CD4 counts compared to those in steady state. However, haemoglobin levels did not show a statistically significant difference between the crisis state (9.43 ± 1.98 g/dL) and steady state (10.08 ± 1.60 g/dL; *P *= 0.231). Gender-based analysis indicated no significant difference in CD4 + counts (*P *= 0.403) ++ or haemoglobin levels (*P *= 0.542) between male and female patients. Age-related analysis showed the highest mean CD4 + count among the 31–40-year age group (1151.80 ± 626.06 cells/µL) and the lowest in patients aged 40 years and above (601.00 ± 0.00 cells/µL). Correlation analysis demonstrated weak and non-significant relationships between CD4 + counts and haemoglobin levels (*r *= −0.095, *P *= 0.530), gender (*r *= −0.126, *P *= 0.403), and age (*r *= 0.193, *P *= 0.198). Conclusion: These findings highlight significant reduced CD4 levels in SCD patients during crises, underscoring the need for regular immunological monitoring.

## Introduction

Sickle cell disease (SCD) is a chronic genetic disorder of haemoglobin synthesis characterized by the presence of the abnormal haemoglobin S [1]. Patients with vaso-occlusive crisis (VOC) present with moderate to severe pain, which has variable intensity and frequency [2]. This leads to sickling of red blood cells, chronic haemolysis, and vaso-occlusive crisis that can cause multi-organ damage and early mortality [3]. The SCD is particularly prevalent in sub-Saharan Africa, where Nigeria carries the highest burden, with approximately 150,000 children born annually with the condition [4, 5]. Apart from its haematological manifestations, SCD also affects the immune system, rendering patients more vulnerable to infections [6]. Furthermore, immunological alterations, especially in T-cell-mediated responses, are increasingly being recognized as significant contributors to disease severity and outcomes in SCD [7].

Several studies have documented immune abnormalities in SCD patients, with altered levels of CD4 + and CD8 + T-lymphocytes being frequently reported [8, 9]. These T-cells are essential for immune regulation and pathogen defence. Reports have shown increased activation and turnover of CD4 + T-cells, possibly due to chronic inflammation and antigenic stimulation from repeated infections and haemolysis [1, 10]. However, findings on CD4 + T-cell counts have been inconsistent across various geographical locations and population groups. For example, while some studies indicated elevated CD4 + counts suggestive of immune activation [11], others reported reduced levels possibly linked to immune exhaustion or dysregulation [9]. Such variability underscores the need for context-specific investigations into the immunological profiles of SCD patients.

Despite numerous studies in other parts of Nigeria and globally, data on CD4 + T-lymphocyte counts in adult SCD patients in North-western Nigeria are limited. Most previous researches have focused on paediatric populations or regions with different environmental and healthcare settings. This gap hinders accurate risk stratification and the development of tailored interventions for adult SCD patients in this part of the country. Understanding the immune status of these patients, especially their CD4 + profiles, is vital for preventing and managing infectious complications and improving overall care outcomes [12, 13].

This study aims to evaluate CD4 + T-lymphocyte levels among adult SCD patients attending Specialist Hospital Sokoto using a flow cytometric approach. By comparing the CD4 + counts of SCD patients with those of age- and sex-matched healthy controls, this research seeks to provide baseline immunological data for the adult SCD population in this region. The findings are expected to contribute valuable insights toward optimizing immunological monitoring and designing evidence-based clinical guidelines for managing SCD patients in Northwestern Nigeria.

## Materials and methods

### Study area

The research was conducted at Specialist Hospital Sokoto, situated in the Sokoto South LGA of Sokoto State. The hospital has a capacity of 448 beds and serves between 11,000 to 13,000 patients per month on average. Sokoto is positioned in the Sahel savannah of northwestern Nigeria, at longitude 5° 14’ East and latitude 13° 14’ North. It borders the Niger Republic to the north, Kebbi State to the southwest, and Zamfara to the east. The state encompasses an area of roughly 32,000 square kilometers, with a population of 4,602,298 million [10]. According to the 2014 national population census, the population totalled 3.7 million with an annual growth rate of 3.0% [10]. Sokoto state experiences a semi-arid climate, characterized by Sudan Savannah vegetation, with annual rainfall between 500–1300 mm and temperatures ranging from 15°C to above 40°C on warmer days [11].

### Study design

A descriptive cross-sectional study was conducted to assess CD4 + T-lymphocyte counts among adult patients with sickle cell anaemia in a steady state. The study population comprised individuals aged 18 to 50 years, including 30 adult SCA patients (HbSS genotype) who met the criteria for a steady state defined as having stable haematocrit and haemoglobin values over a period of 2 to 3 months, with no signs or symptoms of HIV infection, over infections, pain crises, or other acute episodes. This status was confirmed through a detailed medical history and comprehensive physical examination. A control group of 30 prospective, apparently healthy blood donors with normal haemoglobin genotype (HbAA) was recruited. Controls were matched to the SCA patients by age (±3 years) and sex to minimize potential confounding variables. Socio-demographic data were collected using a structured questionnaire, and laboratory investigations, including CD4 + T-lymphocyte count assessments, were performed for all participants.

### Study population

This study included confirmed adult sickle cell disease patients attending the Sickle Cell Clinic of Specialist Hospital Sokoto. Participants were enrolled only in a stable state, defined as at least four weeks without a VOC, acute illness, hospitalization, or blood transfusion. VOC was defined as acute bone or joint pain requiring clinical evaluation and not explained by infection or trauma. Age and sex matched, apparently healthy individuals with a confirmed haemoglobin AA genotype served as controls.

### Sample size determination

The sample size was calculated using single population proportion formula (1967);


$$n=\frac{z^{2}\times p\times q}{d^{2}}$$

Where;

*n*: Minimum number of samples required

*Z*: Standard deviation at 95% confidence interval = 1.96

*p*: Prevalence, 3% which will account for 0.03 [14].

d: Degree of freedom (5%) = 0.05*q*: (1-*p*) =0.97


$$n=\frac{1.96^{2}\times 0.03\times 0\cdot 97}{0.05^{2}}$$

$$n=\frac{3\cdot 8416\times 0\cdot 03\times 0\cdot 97}{0.0025}$$

n=44.7

Approximately 45 samples were collected.

### Sampling technique

A convenience sampling technique was used to recruit eligible participants from the Sickle Cell Clinic and the general outpatient department for controls.

### Inclusion and exclusion criteria

Participants included in the study were adults aged 18 years and above with a confirmed diagnosis of Sickle Cell Disease (HbSS) in a stable state, who had provided informed consent. Individuals were excluded if they had received a blood transfusion within the preceding four weeks, were experiencing an acute illness or sickle cell crisis at the time of recruitment, tested positive for HIV, were pregnant, or were receiving immunosuppressive therapy.

### Sample collection

A total of 4 ml of venous blood was collected from each participant into an ethylenediaminetetraacetic acid (EDTA) container using sterile technique. Samples were gently mixed and transported in cold chain conditions to the laboratory for immediate processing.

### Laboratory analysis

CD4 + T-lymphocyte counts were determined using flow cytometry. The Partec CyFlow^®^ Counter (Sysmex Partec GmbH, Germany), a compact single-platform flow cytometer, was used for absolute CD4 + count determination. The procedure followed the manufacturer’s instructions: Blood samples were mixed with CD4 monoclonal antibodies tagged with fluorochrome and incubated for 15 minutes in the dark. The samples were then lysed and analyzed. Results were expressed as absolute counts of CD4 + cells per microliter (cells/μL) of blood.

### Data analysis

Data obtained were entered into Microsoft Excel and analysed using SPSS version 23.0 (IBM Corp, Armonk, NY). Descriptive statistics such as mean and standard deviation were used to summarize continuous variables. An independent sample *t*-test was used to compare the mean CD4 + T-cell counts between the SCD patients and the control group. A *p*-value of less than 0.05 was considered statistically significant.

## Results

From Table 1, the mean CD4 + T-lymphocyte count was significantly higher in patients in stable state (1068.92 ± 378.59 cells/μL) compared to those in crisis (564.05 ± 111.60 cells/μL), with a t-value of 5.934 and a *p*-value of < 0.001. There was no significant difference in haemoglobin levels between patients in stable state (9.428 ± 1.98 g/dL) and those in crisis (10.081 ± 1.60 g/dL), with a t-value of -1.215 and a *p*-value of 0.231.

**Table 1.** The comparison of CD4 + T-lymphocyte counts and haemoglobin levels between steady state and acute sickle cell crises patients.

GROUPS	**CD4 (**cells/μL)	**HGB** (g/dL)
STEADY STATE	1068.92 ± 378.59	9.428 ± 1.98
IN CRISIS	564.05 ± 111.60	10.081 ± 1.60
*t*-value	5.934	−1.215
*p-value*	<0.001	0.231

As shown in Table 2, there was no significant difference in CD4 + T-lymphocyte counts between male (884.00 ± 884.00 cells/μL) and female patients (788.73 ± 320.733 cells/μL), with a t-value of 0.844 and a *p*-value of 0.403. Additionally, there was no significant difference in haemoglobin levels between male (9.567 ± 1.99 g/dL) and female patients (9.900 ± 1.65 g/dL), with a t-value of -0.614 and a *p*-value of 0.542.

**Table 2.** The comparison of CD4 + T-lymphocyte counts and haemoglobin levels between male and female patients.

GROUPS	**CD4 (**cells/μL)	**HGB **(g/dL)
Male	884.00 ± 884.00	9.567 ± 1.99
Female	788.73 ± 320.733	9.900 ± 1.65
*t*-value	0.844	−0.614
*p*-value	0.403	0.542

As indicated in Table 3, there was no significant difference in CD4 + T-lymphocyte counts and haemoglobin levels across the different age groups with a *p*-value of 0.231and 0.665 respectively.

**Table 3.** CD4 + T-lymphocyte counts and haemoglobin levels across different age groups.

GROUPS	CD4 **(**cells/μL)	HGB (g/dL)
18–25	785.00 ± 293.783	9.950 ± 1.79
26–30	852.33 ± 428.105	9.150 ± 1.97
31–40	1151.80 ± 626.062	9.820 ± 1.97
40 and above	601.00 ± 0.00	9.900 ± 0.00
*p-*value	0.231	0.665

Note: The *p*-value for the 40 and above age group is not applicable due to the small sample size (*n* = 1).

There was no significant correlation between CD4 + T-lymphocyte count and haemoglobin levels (r = −0.095, *P* = 0.530) as shown in Table 4. Moreso, the correlation between CD4 + T-lymphocyte count and gender (r = −0.126, *P* = 0.403) was found to be statistically insignificant. Although there was a weak positive correlation between CD4 + T-lymphocyte count and age group, it was found to be statistically insignificant (r = 0.193, *P* = 0.198) as shown Table 4.

**Table 4.** The correlation analysis between CD4 + T-Lymphocyte count and other variables.

		**HGB **(g/dL)	GENDER	AGE (yrs)
CD4 (cells/μL)	Pearson Correlation	−0.095	−0.126	0.193
	Sig. (2 tailed)	0.530	0.403	0.198
	N	46	46	46

## Discussion

This study assessed the CD4 + T-lymphocyte counts in adult sickle cell disease patients attending Specialist Hospital Sokoto and examined variations across clinical states, gender, and age groups. The results revealed a significantly lower mean CD4 + count during vaso-occlusive crises compared to the stable state, while haemoglobin levels showed no significant difference between the two states. These findings highlight the profound impact of acute sickle cell complications on cellular immunity and support the hypothesis that immune suppression is exacerbated during crises. The mean CD4 + T-lymphocyte count in patients during the steady state (1068.92 ± 378.59 cells/μL) was comparable to values reported in similar Nigerian studies [13, 15], suggesting that despite chronic haemolysis and inflammation, some degree of immune function is retained outside of acute episodes. In contrast, the marked reduction during crises (564.05 ± 111.60 cells/μL) aligns with prior findings showing CD4 + lymphopenia associated with increased disease severity, inflammation, and T-cell exhaustion [9, 16]. These changes may be mediated by increased cytokine levels and immune activation during crises, as reflected in elevated interleukin-6 and TNF-α levels [17, 18].

Gender-based analysis did not show statistically significant differences in CD4 + counts or haemoglobin levels. Although females had slightly higher CD4 + counts on average, the difference was not meaningful. This is consistent with earlier studies that found minimal gender-based variation in lymphocyte subsets among SCD populations [15]. Similarly, age-based analysis revealed the highest CD4 + levels among participants aged 31–40 years and the lowest among those 41 years and above, though this variation was also not statistically significant. Some studies have suggested that aging contributes to immune decline in SCD patients due to cumulative oxidative stress, chronic inflammation, and organ dysfunction [19, 20]. Correlation analyses further demonstrated weak and non-significant relationships between CD4 + count and haemoglobin, age, or gender. This suggests that while these variables may influence immune status, other factors such as infection history, hydroxyurea therapy, transfusion frequency, or genetic polymorphisms could have stronger impacts [21]. The lack of strong correlations also underscores the complex and multifactorial nature of immune dysregulation in SCD. Overall, the results of this study reinforce previous evidence that immune competence is compromised in SCD, particularly during vaso-occlusive crises. The findings support the need for routine immunological monitoring of SCD patients and may guide clinicians in initiating prophylactic treatments, vaccinations, or closer surveillance during crisis episodes. Additionally, this study contributes valuable regional data on the immunological profiles of adult SCD patients in Northwestern Nigeria, a population that has been underrepresented in previous literature [22].

Our study has some limitations. This is cross sectional study, which involved a single timepoint for each patient. Moreover, the study only measured CD4, future studies that assessing inflammatory markers such as C‑reactive protein, white blood cells, CD8 and platelets count and cytokines assay would give a robust findings.

## Conclusion

This study demonstrated that CD4 + T-lymphocyte counts are significantly reduced in adult sickle cell disease patients during vaso-occlusive crises compared to their steady-state levels. Although haemoglobin levels also decreased during crises, the difference was not statistically significant. No significant associations were found between CD4 + counts and variables such as gender, age, or haemoglobin concentration. These findings underscore the critical role of immune suppression during acute episodes in SCD and highlight the need for immunological monitoring in routine care. The results provide valuable baseline data on adult SCD patients in Northwestern Nigeria a population underrepresented in existing literature and add to the growing body of evidence on immune dysregulation in SCD.

## Ethical considerations

Ethical approval for this study was obtained from the Ethical Committee of Specialist Hospital Sokoto (SHS/SUB/133/VOL-1). Informed consent was obtained from all participants after a thorough explanation of the study objectives, procedures, and potential risks. Participation was voluntary, and patients were allowed to withdraw at any time they wish without compromising their access to care. Confidentiality was strictly maintained throughout the study.

## Conflict of interest

All authors declare that there is no conflict of interest regarding this manuscript.

## Author contributions

Abubakar Usman Babangida(Conceptualization [Lead], Data curation [Lead], Methodology [Lead], Supervision [Equal], Writing—original draft [Equal], Writing—review & editing [Equal]), Abubakar Gulma Hassan(Conceptualization [Equal], Formal analysis [Equal], Writing—original draft [Equal]), Kabir Hamid Magaji(Conceptualization [Equal], Formal analysis [Equal], Methodology [Equal], Visualization [Equal]), Adamu Muhammmad Ibrahim Ibrahim(Conceptualization [Equal], Data curation [Equal], Writing—original draft [Equal], Writing—review & editing [Equal]), Shuabu Musa Saidu(Writing—original draft [Equal], Writing—review & editing [Equal]), Jomar Aban L.(Validation [Equal], Writing—original draft [Equal], Writing—review & editing [Equal]), Jerico Ogaya Bautista (Writing—original draft [Equal], Writing—review & editing [Equal]), Kristine Gacutno Joy(Writing—original draft [Equal], Writing—review & editing [Equal]), Angelica Gacutno-Evardone Joyce (Methodology [Equal], Writing—original draft [Equal], Writing—review & editing [Supporting]), Carina Barroso Joane V.(Writing—original draft [Equal], Writing—review & editing [Equal]), Pearl Irish De Paz V. (Validation [Equal], Writing—original draft [Equal]), Mohamed Mustaf Ahmed(Visualization [Equal], Writing—original draft [Equal], Writing—review & editing [Equal]), and Sani Muhammed(Methodology [Equal], Writing—original draft [Equal], Writing—review & editing [Equal]), Don Lucero-Prisno-III Eliseo(Supervision [Equal], Writing—review & editing [Equal]).

## Funding

This article did not receive any funding from any governmental, commercial or not-for-profit organisation.

## Availability of data and materials

All relevant data are available from the corresponding author on request [amuhammadibrahim37@gmail.com].

## Data Availability

The data underlying this article will be shared on reasonable request to the corresponding author.
